# Mechanism of Activation of AMPK by Cordycepin

**DOI:** 10.1016/j.chembiol.2020.01.004

**Published:** 2020-02-20

**Authors:** Simon A. Hawley, Fiona A. Ross, Fiona M. Russell, Abdelmadjid Atrih, Douglas J. Lamont, D. Grahame Hardie

**Affiliations:** 1 Division of Cell Signalling & Immunology, School of Life Sciences, University of Dundee, Dow Street, Dundee DD1 5EH, UK; 2 Fingerprints Proteomics Facility, School of Life Sciences, University of Dundee, Dundee, UK

**Keywords:** AMP-activated protein kinase, AMPK, cordycepin, metabolism, 3′-deoxyadenosine, mechanism

## Abstract

Cordycepin (3′-deoxyadenosine) is a major bioactive agent in *Cordyceps militaris*, a fungus used in traditional Chinese medicine. It has been proposed to have many beneficial metabolic effects by activating AMP-activated protein kinase (AMPK), but the mechanism of activation remained uncertain. We report that cordycepin enters cells via adenosine transporters and is converted by cellular metabolism into mono-, di-, and triphosphates, which at high cordycepin concentrations can almost replace cellular adenine nucleotides. AMPK activation by cordycepin in intact cells correlates with the content of cordycepin monophosphate and not other cordycepin or adenine nucleotides. Genetic knockout of AMPK sensitizes cells to the cytotoxic effects of cordycepin. In cell-free assays, cordycepin monophosphate mimics all three effects of AMP on AMPK, while activation in cells is blocked by a γ-subunit mutation that prevents activation by AMP. Thus, cordycepin is a pro-drug that activates AMPK by being converted by cellular metabolism into the AMP analog cordycepin monophosphate.

## Highlights

• Cordycepin (100 μM) activates AMPK in human cells; higher concentrations are toxic
• Cordycepin is taken up into cells and converted into mono-, di-, and triphosphates
• AMPK activation correlates with the cellular content of cordycepin monophosphate
• Cordycepin monophosphate mimics all three effects of AMP on AMPK in cell-free assays

## Graphical Abstract

**Figure:**
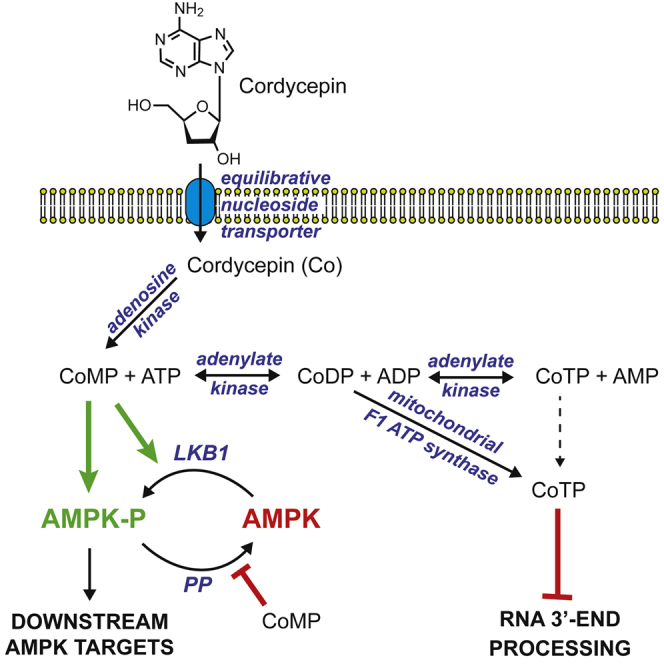

## Teaser

Cordycepin is a major bioactive component of *Cordyceps militaris*, a parasitic fungus much prized in traditional Chinese medicine. Hawley et al. report that it is taken into cells and converted by the metabolism into mono-, di-, and triphosphates. Cordycepin monophosphate activates AMPK by mimicking multiple effects of its natural activator, AMP.

## Introduction

Cordycepin (3′-deoxyadenosine) is an adenosine analog derived from *Cordyceps militaris*, a parasitic fungus that infects insect larvae and is highly prized in traditional Chinese medicine (Tuli et al., 2013). Cordycepin is taken up into cells and converted to mono-, di-, and triphosphates (Klenow, 1963); since it lacks a 3′-hydroxyl group, if incorporated into RNA it would cause chain termination. Indeed, cordycepin inhibits RNA synthesis in cells, as well as inhibiting RNA polymerases I, II, and III and (more potently) poly(A) polymerases in cell-free assays (Muller et al., 1977). In budding yeast, cordycepin reduces the amount of poly(A)^+^ RNA without affecting rRNA or tRNA, while mutations in the poly(A) polymerase Pap1 have similar effects on global gene expression (Holbein et al., 2009). In mammalian cells, cordycepin reduces the poly(A) tail lengths of some, but not all, mRNAs (Wong et al., 2010). These results indicate that a major mode of cordycepin action is to inhibit 3′ end processing of mRNAs.

Cordycepin has also been reported to activate AMP-activated protein kinase (AMPK) (Guo et al., 2010, Wong et al., 2010). This has been proposed to be how the compound prevents hyperlipidemia induced by high-fat diet in hamsters (Guo et al., 2010), inhibits the mammalian target-of-rapamycin complex-1 (mTORC1) (Wong et al., 2010), downregulates mTORC1 function and HIF-1α expression in tumor cells (Wu et al., 2014b), inhibits TNF-α production in macrophages (Zhang et al., 2014), activates autophagy (Marcelo et al., 2019), inhibits senescence and radiation ulcers in mouse skin and intestine (Wang et al., 2019), and inhibits survival, migration, and invasion of lung cancer cells (Wei et al., 2019). In some of these studies the evidence for involvement of AMPK relied on the inhibitor compound C, which has very poor selectivity for AMPK (Bain et al., 2007), and in none was the detailed mechanism for AMPK activation established.

AMPK is a sensor of cellular energy status occurring as heterotrimeric complexes comprising a catalytic α subunit and regulatory β and γ subunits. In mammals, each subunit has alternate isoforms (α1/α2; β1/β2; γ1/γ2/γ3) encoded by distinct genes (Ross et al., 2016b, Lin and Hardie, 2017). AMPK is significantly active only after phosphorylation at Thr172 within the kinase domain by upstream kinases, especially the tumor suppressor LKB1. Binding of AMP to the γ subunit activates AMPK by three complementary mechanisms: (1) allosteric activation (Yeh et al., 1980), (2) promotion of Thr172 phosphorylation by upstream kinases (Hawley et al., 1996, Oakhill et al., 2010, Ross et al., 2016a), (3) inhibition of Thr172 dephosphorylation by protein phosphatases (Davies et al., 1995). Although allosteric activation is caused only by AMP, effects (2) and (3) are mimicked by higher concentrations of ADP (Oakhill et al., 2011, Xiao et al., 2011, Gowans et al., 2013, Ross et al., 2016a). These activating effects are antagonized by ATP, so that AMPK is activated by increases in AMP:ATP and ADP:ATP ratios, which occur whenever cellular energy is compromised. AMPK then acts to restore energy homeostasis by switching on catabolic pathways, while switching off most anabolic processes (Ross et al., 2016b, Lin and Hardie, 2017).

Because cordycepin 5′-monophosphate (CoMP) is a close analog of AMP, it seemed likely that it would mimic effects of AMP on AMPK. Indeed, binding of CoMP to the AMPK-α1 and -γ1 subunits has been modeled by molecular docking (Wang et al., 2010, Wang et al., 2019), while cordycepin was reported to bind to the isolated AMPK-γ1 subunit (Wu et al., 2014a) and CoMP to allosterically activate AMPK (Wang et al., 2010). However, no detailed studies of the molecular mechanism(s) by which cordycepin activates AMPK have been reported until now.

## Results

### Cordycepin Activates AMPK in Intact Cells

**Figure 1:**
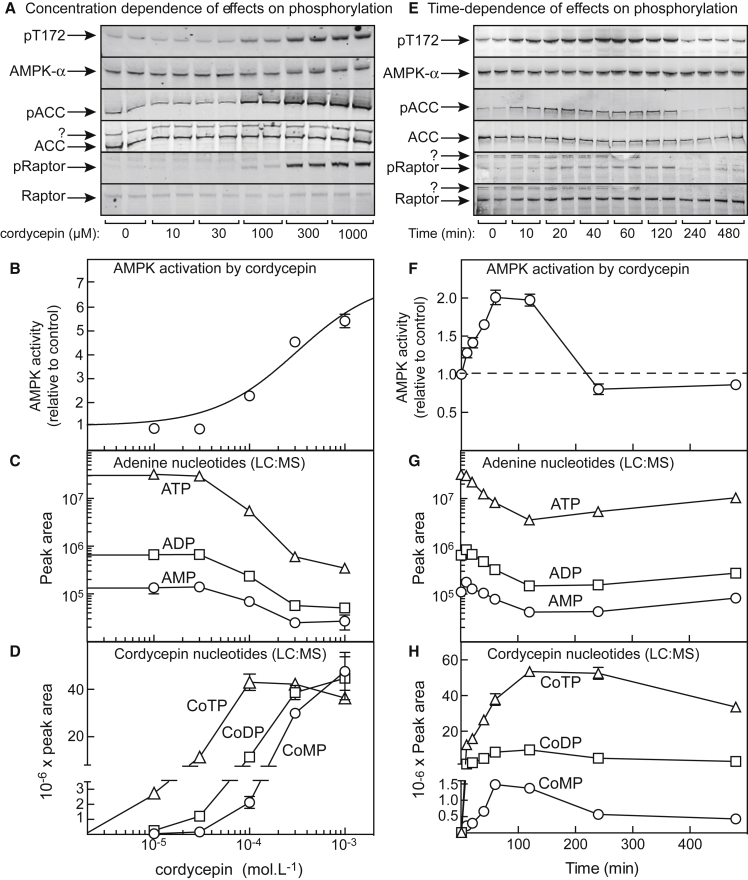
AMPK Activation and Nucleotide Contents in HepG2 Cells Treated with Cordycepin (A–D) Changes induced by incubation with cordycepin for 1 h. (A) Phosphorylation of AMPK, ACC, and Raptor. (B) AMPK activity in immunoprecipitates. (C) Cellular content of adenine nucleotides (note logarithmic scale on y axis). (D) Cellular content of cordycepin nucleotides. (E–H) Time courses of changes induced by incubation with 100 μM cordycepin. (E) Phosphorylation of AMPK, ACC, and Raptor. (F) AMPK activity in immunoprecipitates. (G) Cellular content of adenine nucleotides. (H) Cellular content of cordycepin nucleotides. For (B), results are expressed relative to control and were fitted to the following equation: activity = 1 + (((activation − 1) × [AMP])/(EC_50_ + [AMP])). The curve was generated with best-fit parameters mentioned in the text. Results are means ± SEM; n = 2 in (A) and (E), n = 4 to 6 in (B), n = 4 in (F), n = 3 in (C), (D), (G), and (H). In (A) and (E), “?” indicates non-specific bands detected by the probes used. See also Figure S1.

Incubation of HepG2 cells with cordycepin at 100 μM and above for 1 h increased phosphorylation of Thr172 on AMPK itself and of AMPK sites on two downstream targets, acetyl-coenzyme A carboxylase (ACC1, Ser80) and Raptor (Ser792) (Figure 1A). The kinase activity of AMPK also increased by a maximum of 7 ± 1-fold (±SEM), with a half-maximal effect (EC_50_) at 310 ± 60 μM (Figure 1B). No activation was evident at 30 μM, although it has been reported that cordycepin activated AMPK in HepG2 cells at concentrations as low as 1 μM, using incubations in medium without serum but with 0.02% (w/v) bovine serum albumin (BSA) (Guo et al., 2010). When we repeated our experiments under those conditions, cordycepin was indeed much more potent (Figure S1A). Thus, the potency of cordycepin is enhanced in serum-free medium, most likely due to its sequestration by some component of serum. Consistent with this, when cordycepin (100 μM) was incubated with cell medium and centrifugally filtered through a membrane with a cutoff of 3 kDa (which would allow cordycepin through, but not proteins), the recovery of cordycepin was 4- to 5-fold lower when 10% serum had been added to the medium, compared with medium plus 0.02% BSA or medium alone (Figure S1B). Note that all of the papers cited above (other than Guo et al. (2010)) that studied effects of cordycepin on AMPK included serum and used cordycepin at 10–200 μM. We believe that serum-free conditions are less physiologically relevant, so we included serum in all other experiments.

We next monitored the cellular contents of adenine and cordycepin nucleotides by liquid chromatography-mass spectrometry (LC-MS). At cordycepin concentrations above 30 μM, levels of ATP, ADP, and AMP progressively declined (Figure 1C). Remarkably, at 1 mM cordycepin the ATP content had dropped by 100-fold (note logarithmic scale), although the decreases in ADP (14-fold) and AMP (5-fold) were smaller. Over the same concentration range, the levels of cordycepin triphosphate (CoTP), cordycepin diphosphate (CoDP), and CoMP increased (Figure 1D), with CoTP accumulating at lower cordycepin concentrations and CoDP and CoMP only at higher concentrations. AMPK activity correlated best with CoMP: for example, neither AMPK activation nor CoMP formation was observed at 30 μM cordycepin or below, although both CoDP and CoTP had increased (compare Figures 1B and 1D). Figure S1C shows the same data plotted as cellular energy charge for either adenine or cordycepin nucleotides. Although adenine nucleotide energy charge did decrease slightly at the highest cordycepin concentrations, the cordycepin nucleotide energy charge decreased much more markedly and showed a much better negative correlation with AMPK activity.

Using standards for reference, the recovery of CoTP during perchloric acid extraction and LC-MS (see STAR Methods) was lower (57 ± 3%) than that of ATP, so the finding that the peak area of CoTP after incubation of cells with cordycepin at 100 μM and above for 1 h (4 × 10^7^, Figure 1D) was actually higher than that of ATP in cells incubated without cordycepin (3 × 10^7^, Figure 1C) indicated (remarkably) that CoTP was almost completely replacing ATP within the cells.

We next examined the time course of AMPK activation and nucleotide changes at 100 μM cordycepin. The increases in phosphorylation and activation of AMPK were transient, peaking at ≈2-fold at 1 h and returning to baseline by 4 h (Figures 1E and 1F). Decreases in adenine nucleotides (Figure 1G) were also transient, peaking at 2 h and then slowly reversing, although recovery was incomplete by 8 h. Cordycepin nucleotides also reached a maximum at 1–2 h (Figure 1H) and then slowly declined. Once again, the best correlation with AMPK activity was with CoMP, since both AMPK activity and CoMP had almost returned to baseline by 4 h, whereas the levels of CoDP and CoTP remained elevated up to 8 h.

The reversal of the effects of cordycepin after 2 h was likely because cordycepin was being taken up and metabolized by the cells. Measurements of cordycepin in the medium revealed that it had declined by 70% by 2 h and had almost disappeared by 4 h; the half-life of cordycepin in the medium was about 80 min (Figure S1D).

### Effects of Cordycepin on Cell Viability

**Figure 2:**
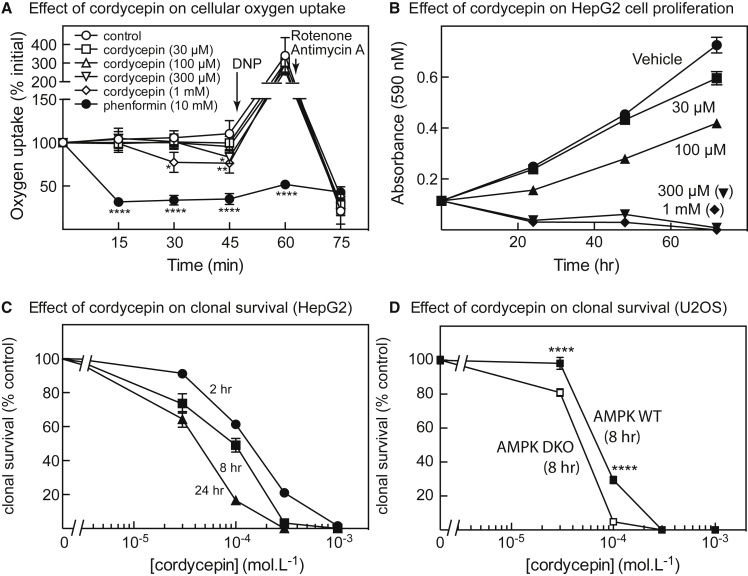
Effects of Cordycepin on Oxygen Uptake, Cell Proliferation, and Viability (A) Changes in oxygen uptake following addition of cordycepin or phenformin to HepG2 cells. At the points shown by arrows, 2,4-dinitrophenol (DNP, 100 μM) or rotenone (2 μM) plus antimycin A (1 μM) were added. Results are means ± SD (n = 6 to 8); results labeled with asterisks are significantly different from control at the same time point (*p < 0.05, **p < 0.01, ****p < 0.0001). (B) Effect of cordycepin on cell proliferation assessed by 3-(4,5-dimethylthiazol-2-yl)-2,5-diphenyltetrazolium bromide assays; results are means ± SEM (n = 6). (C) Effect of incubation of HepG2 cells with cordycepin for the indicated time on cell viability, assessed by clonal survival assays; results are means ± SEM (n = 3). (D) Effect of incubation of wild-type (WT) and AMPK DKO U2OS cells with cordycepin for 8 h on clonal survival; results are means ± SEM (n = 3). Results significantly different for WT and DKO cells are indicated by asterisks (****p < 0.0001). See also Figure S2.

The results discussed above suggested, remarkably, that at cordycepin concentrations of 300 μM and above, cordycepin nucleotides almost completely replaced adenine nucleotides in the cells, at least transiently. Surprisingly, the cells appeared to remain viable, at least in the short term. For example, Figure 2A shows that, unlike the complex I inhibitor phenformin, cordycepin had only minor effects on basal oxygen uptake, with modest inhibition by 300 μM after 45 min and by 1 mM after 30 and 45 min. There were no significant effects on maximal oxygen uptake measured after addition of the uncoupler 2,4-dinitrophenol or on residual oxygen uptake after subsequent addition of the respiratory chain inhibitors rotenone and antimycin A.

To assess longer-term effects of cordycepin, we examined its effects on cell proliferation and clonal survival. Figure 2B shows that cordycepin at 30 μM had only very small effects on proliferation after 72 h, while at 100 μM it caused a 30% decrease. However, 300 μM and 1 mM cordycepin completely prevented cell proliferation. At 300 μM and 1 mM, cordycepin also caused almost complete death of HepG2 cells in clonal survival assays, whereas 100 μM had only partial effects (Figure 2C). The effects depended on the incubation time; half-maximal effects on cell survival after 2, 8, and 24 h treatment were at 130, 70, and 35 μM, respectively.

To assess whether AMPK provided protection against cell death induced by cordycepin, we utilized double-knockout (AMPK DKO) human osteosarcoma (U2OS) cells in which both the α1 and the α2 catalytic subunit isoforms had been knocked out using CRISPR. Figure S2A shows AMPK activation in wild-type (WT) U2OS cells by different concentrations of cordycepin, compared with treatment with phenformin or starvation for both glucose and glutamine, these being treatments that activate AMPK by AMP-dependent mechanisms (Hawley et al., 2010, Zhang et al., 2017). To confirm AMPK knockout, Figure S2B shows that AMPK could not be detected in the DKO cells using either anti-α1 or pan-α antibodies, while increased phosphorylation of ACC in response to H_2_O_2_ was also completely abolished. Figure 2D shows that DKO cells were significantly more sensitive to cordycepin in clonal survival assays than WT cells, with half-maximal effects at 44 ± 2 and 81 ± 4 μM, respectively.

### Multiple Effects of CoMP on AMPK in Cell-Free Assays

**Figure 3:**
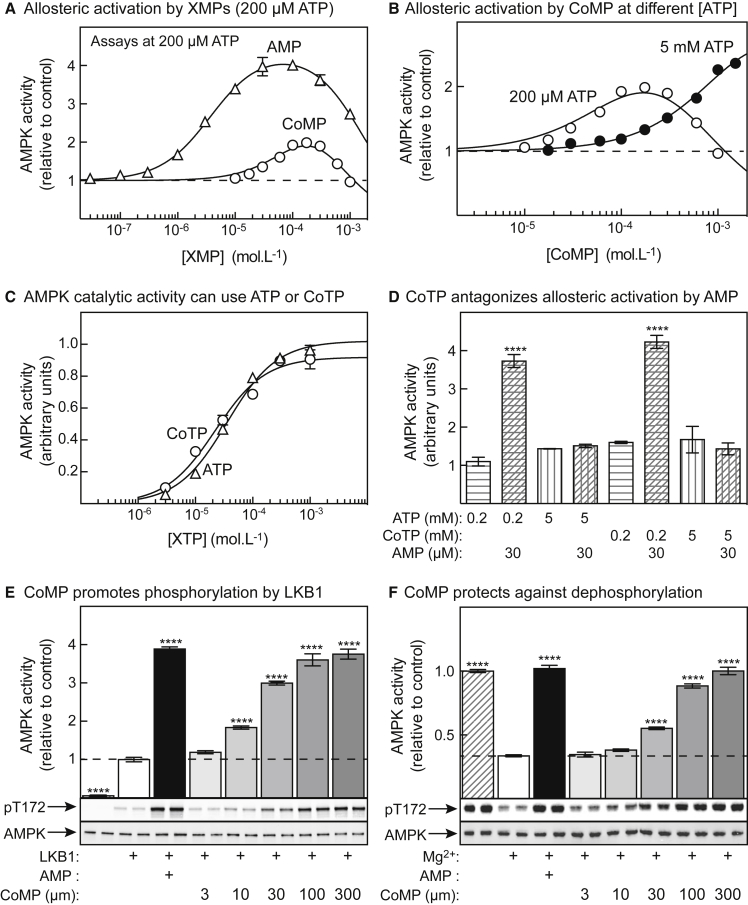
Effects of Cordycepin Nucleotides on AMPK Function in Cell-Free Assays (A) Allosteric activation of AMPK (purified from rat liver) by AMP and CoMP at 200 μM ATP. Results (means ± SD, n = 3) are expressed relative to the activity without AMP/CoMP and were fitted to the following equation: activity = 1 + ((activation − 1) × X)/(EC_50_ + X) − ((activation × X)/(IC_50_ + X)), where activation is maximal activation, X is AMP/CoMP concentration, and EC_50_/IC_50_ is the concentration giving half-maximal activation/inactivation. Curves were drawn with best-fit parameters given in the text. (B) As in (A), but assays were also performed at 5 mM ATP. (C) Phosphorylation of a GST-ACC1 fusion by a human α2β2γ1 complex (phosphorylated on Thr172 using CaMKK2) using ATP or CoTP as co-substrate; phosphorylation was quantified by western blotting using anti-pACC antibody. Mg^2+^ was kept at a constant 4.8 mM excess above [ATP]/[CoTP]. Results (means ± SEM, n = 2) were fitted to the Michaelis-Menten equation: activity = V_max_ × X/(K_m_ + X), where X is ATP/CoTP concentration. Curves were generated using the K_m_ values in the text and estimated V_max_ of 1.02 for ATP and 0.92 for CoTP. (D) Activation of human α2β2γ1 complex by 30 μM AMP when the assays contained 200 μM or 5 mM ATP/CoTP. (E) Promotion of Thr172 phosphorylation by 200 μM AMP and varying CoMP, using human α2β2γ1 complex. (F) Inhibition of Thr172 dephosphorylation by 200 μM AMP and varying CoMP, using purified rat liver kinase. Results significantly different from controls are indicated by asterisks (****p < 0.0001).

Figure 3A shows allosteric activation of purified rat liver AMPK by AMP and CoMP in cell-free assays conducted at 200 μM ATP. Under these conditions, AMP activated 4.4 ± 0.1-fold, with an EC_50_ of 3.8 ± 0.2 μM. At higher concentrations, AMP inhibits AMPK due to binding at the catalytic site (Gowans et al., 2013); the IC_50_ (concentration giving half-maximal inhibition) in this study was 1.6 ± 0.1 mM. CoMP gave a similar bell-shaped curve, although because the activating and inhibitory phases were not as well separated as with AMP, a high degree of uncertainty in best-fit parameters was obtained unless the maximal activation parameter was constrained. If it was constrained to a maximum of 4.4-fold (as obtained for AMP), CoMP activated and inactivated with estimated EC_50_ and IC_50_ values of 120 ± 50 and 500 ± 180 μM, respectively. Thus, CoMP is ≈30-fold less potent as an allosteric activator than AMP.

Figure 3B compares allosteric activation by CoMP at 200 μM ATP and at a more physiologically relevant ATP concentration of 5 mM. As expected, the curve was shifted rightward at 5 mM ATP; EC_50_ increased from 120 μM to 1.4 mM (maximal activation constrained to 4.4-fold). Thus, ATP competes with CoMP for binding at the activating site(s).

Since CoTP appeared to almost replace ATP in cells incubated in high cordycepin concentrations (Figure 1), we tested whether the catalytic domain of AMPK would utilize CoTP as a phosphate donor in place of ATP. Strikingly, bacterially expressed human α2β2γ1 complex could use either ATP or CoTP as phosphate donor with very similar kinetics (Figure 3C), the K_m_ values being 36 ± 3 μM for ATP and 22 ± 3 μM for CoTP. AMP also caused a similar 4-fold activation when the kinase was assayed using either 200 μM ATP or CoTP as substrate, but this activation was abolished when the ATP or CoTP concentrations were increased to 5 mM (Figure 3D). Thus, at high concentrations, both ATP and CoTP antagonize allosteric activation by AMP.

Like AMP, CoMP promoted activation and Thr172 phosphorylation of AMPK by LKB1 (Figure 3E), while also protecting against inactivation and Thr172 dephosphorylation by the protein phosphatase PP2Cα (Figure 3F). Fitting of AMPK activity in Figure 3F as a function of CoMP concentration yielded an EC_50_ of 68 ± 12 μM. While lower than the EC_50_ obtained for allosteric activation (120 μM), the latter was measured in the presence of 200 μM ATP, which would have competed with CoMP for binding to the γ subunit.

### Mechanism for AMPK Activation by Cordycepin in Intact Cells

**Figure 4:**
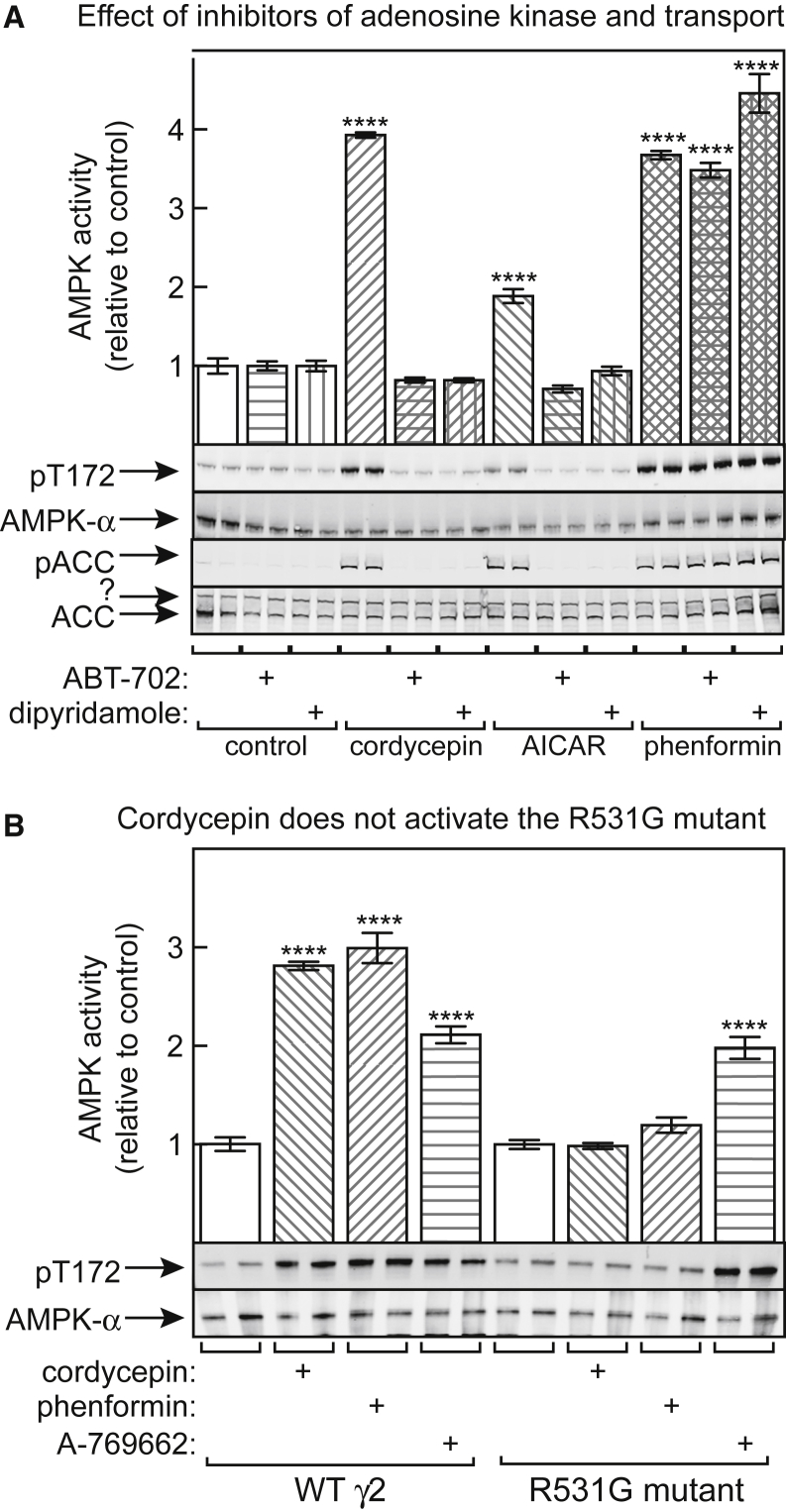
Detailed Mechanism for AMPK Activation by Cordycepin in Intact Cells (A) HepG2 cells were incubated with 300 μM cordycepin, 3 mM AICAR, or 10 mM phenformin for 1 h in the presence or absence of 1 μM ABT-702 or 0.5 μM dipyridamole. AMPK activity was measured in anti-AMPK-α immunoprecipitates (top) and phosphorylation of AMPK and ACC was analyzed in duplicate samples by western blotting. Results in the top graph are means ± SEM (n = 3); asterisks indicate results significantly different from controls without cordycepin, AICAR, or phenformin. (B) HepG2 cells were transiently transfected with DNAs encoding FLAG-tagged wild type (WT) AMPK-γ2 or an R531G mutant. Cells were treated for 1 h ± 300 μM cordycepin, 10 mM phenformin, or 300 μM A-769662, and AMPK activity in anti-FLAG immunoprecipitates was determined; asterisks indicate results significantly different from controls without cordycepin, phenformin, or A-769662 (****p < 0.0001).

We hypothesized that cordycepin enters cells via adenosine transporters and is then converted to CoMP by adenosine kinase. To test this, we made use of dipyridamole and ABT-702, which are inhibitors of Equilibrative Nucleoside Transporters (ENT1 and ENT2; Pastor-Anglada and Perez-Torras, 2018) and adenosine kinase (Jarvis et al., 2000), respectively. Figure 4A shows that both inhibitors blocked the effect of cordycepin to activate and phosphorylate AMPK. As expected, they also blocked the effects of AICA riboside (which is taken up by ENTs; Gadalla et al., 2004), but not phenformin (which activates AMPK by inhibiting the respiratory chain; Hawley et al., 2010).

The results in Figure 3 suggested that CoMP mimicked all three effects of AMP on the AMPK system, albeit with lower potency. To confirm that the CoMP effect required binding to the crucial CBS3 site on the γ subunit, we examined the activation of AMPK complexes in HepG2 cells containing transfected γ2 subunits, with either the WT sequence or the R531G mutation. Arg531 is involved in binding of the phosphate group of AMP to the CBS3 site, and we have shown that this mutation renders AMPK insensitive to AMP (Hawley et al., 2010). Figure 4B shows that AMPK complexes containing WT γ2 were activated similarly by cordycepin, phenformin, and A-769662. However, although complexes containing the R531G mutant were still activated by A-769662 (which, unlike AMP, binds at the ADaM site; Langendorf and Kemp, 2015), they were not activated by phenformin (which increases cellular AMP; Hawley et al., 2010) or cordycepin.

## Discussion

Although it was shown many years ago that cordycepin is converted inside cells into mono-, di-, and triphosphates (Klenow, 1963), we have now quantified by LC-MS the appearance of CoMP, CoDP, and CoTP and the disappearance of AMP, ADP, and ATP, as functions of cordycepin concentration and time. Remarkably, incubation of cells with concentrations of cordycepin above 100 μM caused CoTP, CoDP, and CoMP to almost completely replace adenine nucleotides in the cells. Despite this, the cells remained viable in the short term, with only marginal effects on oxygen uptake. Many cellular ATP-utilizing enzymes may be able to utilize CoTP in place of ATP; indeed, we showed this for AMPK itself, which utilized ATP or CoTP as co-substrate with very similar kinetic parameters. Our finding that treating cells with 1 mM cordycepin for 45 min caused only modest reductions in oxygen uptake also suggests that mitochondrial adenine nucleotide translocases and ATP synthases are able to utilize CoDP to generate CoTP.

High cordycepin concentrations (300 μM and 1 mM, Figure 2) are cytotoxic as judged by cell proliferation and clonal survival assays, most likely due to effects on mRNA synthesis or stability. However, 100 μM cordycepin caused a large activation of AMPK, although a large proportion of cells remained viable in survival assays. It is also clear that AMPK activation helps cells to survive treatment with cordycepin, since its cytotoxic effects were more potent in AMPK-null U2OS cells. The mechanism underlying this is not clear, but it may be because AMPK restrains cell growth and proliferation; cells in a more quiescent state may be more resistant to effects of inhibitors of mRNA synthesis or stability.

Our results suggest that cordycepin activates AMPK in cells via uptake by adenosine transporters (ENT1/ENT2) and conversion by adenosine kinase to CoMP, which then acts as an AMP analog. In cell-free assays, CoMP mimicked all three effects of AMP on the AMPK system, although it was less potent. In addition, the activity of AMPK measured in immunoprecipitate kinase assays (which cannot detect allosteric activation) correlated well with the cellular content of CoMP, but not CoDP, CoTP, or any adenine nucleotide, in both concentration-dependence and time-course experiments. AMPK activity also showed a better negative correlation with the energy charge of cordycepin rather than adenine nucleotides. Although correlations do not prove a causal relationship, taken overall our results suggest that cordycepin activates AMPK in intact cells by conversion to cordycepin monophosphate.

The efficacy of cordycepin as a drug *in vivo* is limited by its rapid cellular uptake and metabolism. In mice treated with a single oral dose of cordycepin of 63 mg/kg, a peak plasma concentration of 10 μM was reached after 1.5 h, and the concentration then declined, with a half-life of 2.1 h (Wei et al., 2009). Cordycepin may be primarily metabolized by adenosine deaminase, which deaminates cordycepin with kinetics similar to those of adenosine (Agarwal et al., 1975). Uptake by ENT1/ENT2 and rapid deamination by adenosine deaminase may explain our findings that cordycepin is rapidly removed from the medium by HepG2 cells.

## Significance

**Cordycepin now joins AICAR (**Corton et al., 1995**) and C13 (**Gomez-Galeno et al., 2010**) in the class of AMPK activators that are pro-drugs converted into AMP analogs by cellular metabolism (although C2, which is derived from C13, binds the γ subunit in a different orientation than AMP;** Langendorf et al., 2016**). While cordycepin can be used to activate AMPK in intact cells, it exhibits cytotoxicity at concentrations only slightly higher than those that activate AMPK. This cytotoxicity is AMPK-independent (although AMPK provides some protection against it) and may be due to the known effects of cordycepin on mRNA synthesis and/or stability. This toxicity of cordycepin, and its rapid cellular uptake and metabolism, may limit its clinical utility except perhaps as a cytotoxic drug for cancer therapy. In that scenario, our finding that AMPK protects against cell death induced by cordycepin suggests that its efficacy might be enhanced by addition of an AMPK inhibitor.**

## STAR★Methods

### Key Resources Table

**Table:** 

REAGENT or RESOURCE	SOURCE	IDENTIFIER
**Experimental Models: Cell Lines**		

HepG2	Public Health England	Cat# 85011430 RRID: CVCL_0027
U2OS TRex	(Grumati et al., 2017)	N/A

**Antibodies**		

pT172 (AMPK-α)	Cell Signaling Technology	Cat# 2535; RRID: AB_331250
AMPK-α1 (for immunoprecipitation)	(Woods et al., 1996)	N/A
AMPK-α2 (for immunoprecipitation)	(Woods et al., 1996)	N/A
AMPK-α1 (for Western blotting)	Cell Signaling Technology	Cat# 2532; RRID: AB_330331
AMPK-α (pan-α, for Western blotting)	AbCam	Cat#ab32047; RRID: AB_722764
EZview™ Red anti-FLAG M2 affinity gel	Sigma-Aldrich	Cat# F2426; RRID: AB_2616449
pACC1/pACC2 (S79/S212)	Cell Signaling Technology	Cat# 11818; RRID: AB_2687505
Streptavidin conjugated to 800 nm fluorophore (for detection of total ACC)	Rockland Immunochemicals	Cat# S000-32;
pS792 (Raptor)	Cell Signaling Technology	Cat# 2083; RRID: AB_2249475
Raptor	Cell Signaling Technology	Cat# 2280; RRID: AB_561245
FLAG	Sigma-Aldrich	Cat# F2426; RRID: AB_2616449

**Molecular Biology Kits**		

KOD Hot Start kit	Sigma-Aldrich	Cat# 71842
MTT assay kit	Abcam	Cat# ab211091
Pierce™ Protein Concentrator PES, 3K MWCO, 0.5 mL	ThermoFisher Scientific	Cat# 88512

**Chemicals, Peptides, and Recombinant Proteins**		

2,4-dinitrophenol	Sigma-Aldrich	Cat# D198501
A-769662	(Goransson et al., 2007)	N/A
ABT-702	Tocris	Cat# 2372
AICAR	Abcam	Cat# ab120358
AMPK purified from rat liver	(Gowans et al., 2013)	N/A
antimycin A	Sigma-Aldrich	Cat# A8674
apyrase	Sigma-Aldrich	Cat# A6535
cordycepin	Sigma-Aldrich	Cat# C3394
cordycepin triphosphate	Sigma-Aldrich	Cat# C9137
dipyrimadole	Tocris	Cat# 0681
DMEM base	Sigma-Aldrich	Cat# D5030
Fugene 6	Promega	Cat# E2691
Human AMPK (α1β2γ1, bacterially expressed)	(Hawley et al., 2012)	N/A
Human LKB1:STRAD-α:MO25-α complex	(Zeqiraj et al., 2009)	N/A
Human PP2Cα	(Davies et al., 1995)	N/A
phenformin	Sigma-Aldrich	Cat# P7045
rotenone	Sigma-Aldrich	Cat# R8875

### Lead Contact and Materials Availability

Further information and requests for resources and reagents should be directed to and will be fulfilled by the Lead Contact, Grahame Hardie (d.g.hardie@dundee.ac.uk). All unique/stable reagents generated in this study are available from the Lead Contact with a completed Materials Transfer Agreement, with reasonable compensation for processing and shipping.

### Experimental Model and Subject Details

#### Cell Culture

HepG2 cells (male) were cultured in Minimum Essential Medium (MEM) supplemented with 10% (v/v) FBS, 1% (v/v) non-essential amino acids and 1% (v/v) penicillin/streptomycin. Transient transfection of DNAs encoding FLAG-tagged AMPK-γ2 or an R531G mutant of AMPK-γ2 were carried out 36-48 hr prior to experiments using Fugene 6 according to manufacturers’ instructions. U2OS cells (female) were cultured in Dulbecco’s Modified Eagle’s Medium (DMEM) supplemented with 10% (v/v) FBS and 1% (v/v) penicillin/streptomycin.

### Method Details

#### CRISPR/Cas9 (D10A)-Mediated Knock-Out of AMPK

AMPK-α1^-/-^ -α2^-/-^ U2OS cells were generated using the Cas9 (D10A) double nickase system. Pairs of guide RNAs targeted to exon 4 in both *PRKAA1* and *PRKAA2*, along with screening primers for genotyping, were designed and cloned by Thomas Macartney and are available by contacting MRCPPU Reagents and Services (https://mrcppureagents.dundee.ac.uk). Sense guides were cloned into the puromycin-selectable pBABED puro U6 vector and antisense guides into the Cas9 (D10A) vector pX335. U2OS cells carrying a Flp recombinase target site were transfected with 1 μg of each plasmid using Fugene6 according to manufacturers’ instructions. At 24 and 48 hr after transfection, medium was replaced with fresh medium containing 1 μg/ml puromycin. Medium was replaced with fresh medium with no selection agent and, after 24 hr, the transfection was repeated without selection. At 24 hr later, single cells were sorted into individual wells of a 96-well plate coated with 0.1% gelatin and containing pre-conditioned McCoy’s 5A medium with 20% FBS. Clones were expanded and screened for loss of AMPK protein and activity by Western blotting using anti-AMPK-α and pACC antibody normalised to total ACC detected using streptavidin. Knockout was also confirmed by genomic DNA sequencing.

#### Production of Cordycepin Monophosphate

CoTP (5 μmol) in water was treated with 8 units of apyrase in the presence of 5 mM CaCl_2_ for 45 min at 30°C. The reaction was terminated by addition of ice-cold perchloric acid to 5% final (v/v). The mixture was neutralised, the concentration of CoMP determined by absorbance (260 nm) and purity confirmed by capillary electrophoresis compared to the precursor, CoTP.

#### MTT Assays for Cell Proliferation

After treatment for the specified time, the effect of cordycepin on cell proliferation was determined using the MTT assay kit (Abcam, ab211091) as per manufacturers’ instructions.

#### Clonal Survival Assays

Cells were seeded into 6-well plates at equal density and treated in triplicate at 40% to 60% confluence with vehicle or cordycepin for the indicated time. Cells were trypsinized in 1 ml trypsin:EDTA for 5 min and diluted in 1 ml of complete medium. Cells in control wells were counted using a hemocytometer and 1000 cells from vehicle and cordycepin-treated wells were seeded in triplicate into 10 cm dishes containing 10 ml of medium. The dishes were incubated at 37°C for 10-15 days. On the last day, the medium was decanted, cells fixed with ice-cold methanol for 10 min and stained with 10% v/v Giemsa stain in water for 15 min. The dishes were washed with water and the number of colonies counted manually.

#### Measurement of Cellular Nucleotides by LC:MS

After treatment, cells for nucleotide analysis were lysed in 70% perchloric acid and the acid extracted as described previously (Hawley et al., 2010). The levels of AMP, ADP, ATP, CoMP, CoDP and CoTP were measured using a TSQ Quantiva (with an ion Max NG source) interfaced with an Ultimate 3000 Liquid Chromatography system (ThermoScientific). Separation of all compounds was achieved using a porous graphitic carbon column (HyperCarb 30x1 mm ID 3 mm; Part No: C-35003-031030, Thermo-Scientific) as described previously (Ross et al., 2017) with some modifications. Mobile phase buffer A consisted of 0.3% (v/v) formic acid adjusted to pH 9 with ammonia prior to a 1:10 dilution. Mobile phase buffer B was 80% (v/v) acetonitrile. The column was maintained at a controlled temperature of 40°C and equilibrated with 10% buffer B for 5 min at a constant flow rate of 0.05 mL/min. Aliquots of 1μL of each sample were loaded onto the column and compounds eluted with a linear gradient from 10% buffer B to 12% buffer B within 1 min, then 12% B to 100% B within 2 min; the column was then washed for 4 min with 100% Buffer B. Eluents were sprayed into the TSQ Quantiva using Ion Max NG ion source with ion transfer tube temperature at 350°C and vaporizer temperature 30°C. The TSQ Quantiva was run in negative mode with a spray voltage of 3500, sheath gas 40, aux gas 20 and sweep gas 2. Levels of ATP, ADP and AMP were measured using multiple reactions monitoring mode (MRM) with transitions described previously (Ross et al., 2017). For CoMP, CoDP and CoTP, optimised collision energies and radio frequencies were determined by infusing the pure compounds. Two transitions were used to monitor each of the three compounds, CoMP (330.22>134.01, 330.22>195.11), CoDP (410>256.67, 410>392.0) and CoTP (490>158.89, 490.11>472.11).

To assess the relative recoveries of ATP and CoTP during LC:MS, triplicate 50 μL samples of CoTP or ATP (both 31 mM) were added to 150 μL of 70% perchloric acid and the acid extracted as described above for cell extracts. The resulting aqueous solutions were then adjusted to 5 mM assuming extinction coefficients of 15.4 l/mmol/cm for ATP and 14.5 l/mmol/cm for CoTP. Analysis of these by extracts by capillary electrophoresis (Hawley et al., 2010) showed that ATP or CoTP constituted 99% or 84% of total adenine or cordycepin nucleotides, respectively (purities estimated by LC:MS were identical). Equal volumes of CoTP and ATP were then mixed, diluted 1000-fold and 3 μL analysed by LC:MS as described above for cell extracts. After correction for the fact that proportion of triphosphate was lower in the CoTP samples, the peak areas were found to be 57 ± 3% lower (n = 3) for CoTP versus ATP standards.

#### Measurement of Cordycepin in Cell Medium

The same LC-MS system was used to detect and quantify cordycepin. LC conditions were optimized using a TSKgel Amine-80 Column (100 × 1 mm, ID 5 μm, Part No.0020010, TOSOH Bioscience). Mobile phase buffer A consisted of 0.1% (v/v) formic acid and buffer B was in 80% (v/v) acetonitrile. The column was maintained at 40°C and was equilibrated with 98% buffer B for 5 min at a constant flow rate of 0.05 mL/min. Samples were diluted 1/1500 and aliquots of 4 μL of each sample loaded onto the column. Cordycepin was eluted by decreasing buffer B from 98% to 85% within 2 min and then to 10% within 5 min. Eluents were sprayed into the TSQ Quantiva which was run in positive mode with a spray voltage of 2700, sheath gas 40, aux gas 20, sweep gas 2. Levels of cordycepin were measured using multiple reaction monitoring mode (MRM) with optimized collision energies and radio frequencies previously determined by infusing pure compound. One transition (252 > 136) was used to detect and monitor cordycepin.

#### Measurements of Cellular Oxygen Consumption

Cellular oxygen consumption rate (OCR) was measured using a Seahorse XF24 Extracellular Flux Analyser with 50,000 cells per well, as described previously (Hawley et al., 2010). HepG2 cells were cultured overnight in 24 well plates (50,000 cells/well) in standard medium as above. One hour before the experiment, the medium was replaced with 675 μl of unbuffered medium, pH 7.4: 8.3 g/L DMEM base (Sigma), 2 mM GlutaMax-1, 5 mM glucose, 32 mM NaCl, and 40 μM Phenol Red. Compounds (75 μl) were injected into wells as specified, and OCR continuously measured as described by the manufacturers of the Analyser (Wu et al., 2007).

#### AMPK Assays Using ATP

AMPK (15 ng) purified from rat liver was assayed in solution using the *SAMS* peptide as substrate (Fyffe et al., 2018) in the presence of AMP or CoMP (concentrations specified in Figure legends), and either 5 mM MgCl_2_ and 200 μM [γ-^32^P]ATP or 9.8 mM MgCl_2_ and 5 mM [γ-^32^P]ATP, thus maintaining a constant excess of [Mg^2+^] over [ATP] (Storer and Cornish-Bowden, 1976). Total assay volume was 25 μl.

Endogenous AMPK in crude cell lysates was first immunoprecipitated using an equal mixture of anti-α1 and –α2 antibodies (150 μg protein) by incubation at 4°C for 2 hr on a roller mixer. After extensive washing, the immunoprecipitates were assayed for AMPK activity (50 μg per assay, total assay volume 50 μl) using the *AMARA* peptide (200 μM) as substrate in the presence of 200 μM AMP, 5 mM MgCl_2_ and 200 μM [γ-^32^P]ATP (Fyffe et al., 2018).

#### AMPK Assays Using CoTP

As ^32^P-labelled CoTP was not available, the experiments in Figures 3C and 3D were performed using non-radioactive CoTP and utilized as co-substrate a construct of glutathione-S-transferase fused at the N-terminus of residues 60-94 of rat ACC1 (Scott et al., 2002), which includes the Ser79 phosphorylation site. Bacterially expressed GST-ACC (0.5 μg) was incubated for 10 min in a total volume of 25 μl with bacterially expressed human AMPK (30 ng of α2β2γ1 complex, previously phosphorylated on Thr172 using CaMKK2) with ATP or CoTP as indicated, and sufficient MgCl_2_ to maintain a constant 4.8 mM excess of [Mg^2+^] over [ATP]. Phosphorylation of this substrate was detected using anti-pACC antibody and quantified using a LiCor Odyssey imager.

#### Cell-Free Assays to Study Effects of AMP/CoMP on Thr172 Phosphorylation

These were as described previously (Fyffe et al., 2018) with some modifications. Bacterially expressed human AMPK (α2β2γ1 complex, unphosphorylated on Thr172, 500 ng) was incubated for 10 min in a total volume of 25 μl with 200 μM ATP, 5 μM MgCl_2_ with/without the LKB1:STRAD:MO25 complex (10 ng), in the presence or absence of AMP (200 μM) or cordycepin as indicated. Aliquots were removed for Western blotting and AMPK assays.

#### Cell-Free Assays to Study Effects of AMP/CoMP on Thr172 Dephosphorylation

These were as described previously (Fyffe et al., 2018) with some modifications. Rat liver AMPK (12.5 μg/ml) was incubated in a shaking incubator at 30°C for 10 min in Hepes buffer (50 mM Na Hepes pH 7.4, 150 mM NaCl, 1 mM dithiothreitol, 0.02% (w/v) Brij-35) with MgCl_2_ and sufficient PP2Cα to yield about 70% inactivation in the absence of added nucleotide. AMP (200 μM) or cordycepin were added at concentrations indicated in figures or legends. Aliquots were removed for Western blotting and AMPK assay. Kinase assays were performed immediately after a further 100-fold dilution, which was sufficient to prevent dephosphorylation during the assay.

#### Assays with AMP-Insensitive (R531G) Mutant

HepG2 cells were transiently transfected with DNAs encoding FLAG-tagged wild type AMPK-γ2 or the R531G mutant, using Fugene 6 according to the manufacturers’ instructions. After transfection for 48 hr, cells were treated with various agents as described, and cell lysates prepared (Hawley et al., 2018). FLAG-tagged AMPK was then immunoprecipitated from 150 μg total protein by incubation at 4°C for 2 hr on a roller mixer with 9 μl of EZview Red anti-FLAG M2 affinity gel. After extensive washing, the immunoprecipitates were assayed for AMPK activity (50 μg per assay in a total volume of 50 μl) using the AMARA peptide (200 μM) as substrate in the presence of 200 μM AMP, 5 mM MgCl_2_ and 200 μM γ-^32^P-ATP as described previously (Fyffe et al., 2018).

#### Assessment of Binding of Cordycepin to Serum Components

Cordycepin (100 μM) was incubated in water, MEM, MEM plus 0.02% BSA, or MEM plus 10% (v/v) serum for 30 min at 37°C in a shaking incubator. The mixtures were then passed through a filter that retains molecules with a mass above ≈3 kDa (Pierce™ Protein Concentrator 3K MWCO) by centrifugation (13,000 xg; room temp; 2-5 min). The filter would retain essentially all proteins, but not free cordycepin. The recovery of cordycepin in the filtrate was then determined using its absorbance at 260 nm after correction for values obtained in controls lacking cordycepin.

#### Other Analytical Procedures

SDS-PAGE for AMPK and Raptor was performed using precast Bis-Tris 4–12% gradient polyacrylamide gels in the MOPS buffer system (Invitrogen). SDS-PAGE for ACC was performed using precast Tris-Acetate 3–8% gradient polyacrylamide gels in the Tris-Acetate buffer system (Invitrogen). Proteins were transferred to nitrocellulose membranes using the iBlot2 system (Invitrogen). Membranes were blocked for 1 hr in Li-Cor Odyssey blocking buffer. The membranes were probed with appropriate antibody (0.1–1 μg/ml) in Li-Cor Odyssey blocking buffer, except where the blotting enhancement system was used (Thermo Scientific; as per manufacturers’ instructions). Detection was performed using secondary antibody (1 μg/ml) coupled to IR 680 or IR 800 dye, or IR-streptavidin 800 dye, and the membranes were scanned using the Li-Cor Odyssey IR imager. Protein concentrations were determined by Coomassie Blue binding with bovine serum albumin as standard (Bradford, 1976).

### Quantification and Statistical Analysis

#### Statistical Analysis

Numbers of replicates (“n”) are given in Figure legends. For intact cell experiments, “n” refers to biological replicates, i.e. independent cell cultures. For cell-free assays, “n” refers to the number of independent replicates; where a two-stage assay was used (e.g. Figures 3E and 3F), both stages were performed independently for each replicate. Statistical significances of differences (indicated on Figures using asterisks: *P<0.05, **P<0.01, ***P<0.001, ****P<0.0001) were estimated using GraphPad Prism 6 for Mac OSX, using 1-way or 2-way ANOVA as appropriate, and the Holm-Sidak multiple comparison test.

### Data and Code Availability

The published article includes all datasets generated or analyzed during this study.

## Supplemental Information

Document S1. Figures S1 and S2

Document S2. Article plus Supplemental Information
