# Supplementary material for: Mechanism of Activation of AMPK by Cordycepin
Source: Cell Chem Biol. 2020 Feb 20;27(2):214–222.e4. doi: 10.1016/j.chembiol.2020.01.004 (PMC7031697; doi:10.1016/j.chembiol.2020.01.004)
Supplement: Document S1. Figures S1 and S2 [file mmc1.pdf]

Cell Chemical Biology, Volume *27*

Supplemental Information

Mechanism of Activation of AMPK by Cordycepin

Simon A. Hawley, Fiona A. Ross, Fiona M. Russell, Abdelmadjid Atrih, Douglas J. Lamont, and D. Grahame Hardie

Hawley et al Figure S1

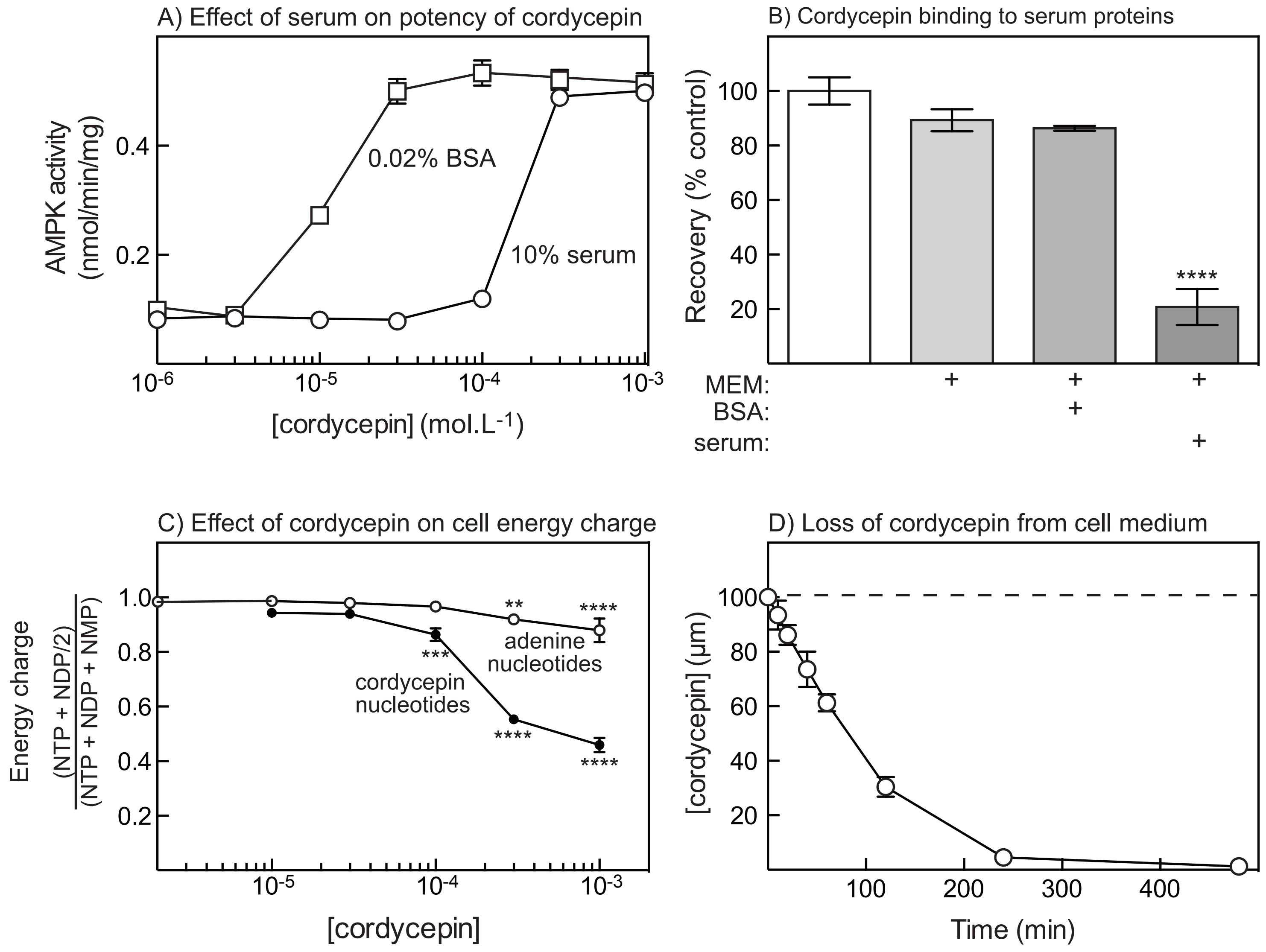

Supplementary Fig. 1 (related to Fig. 1):

(A) Activation of AMPK in HepG2 cells in medium containing 10% fetal calf serum or 0.02% bovine serum albumin. Results are mean ± SD (n = 2).

(B) Cordycepin binds to serum proteins. Results (mean ± SEM, n = 3) show the recovery of cordycepin in the protein-free filtrate when it was incubated for 30 min at 100 µM in water (open bars) or medium (MEM) with or without bovine serum albumin (0.02%, w/v) or fetal calf serum (10% v/v). The mixture was rapidly centrifuged through a filter that retains molecules >3 kDa, and the recovery of cordycepin in the filtrate measured by absorbance at 260 nm. Results are expressed as percantages of the recoveries obtained in water controls, and asterisks denote statistical significance of differences from controls.

(C) Effect of cordycepin concentration on cellular energy charge [(NTP + NDP/2)/(NTP + NDP + NMP)] of adenine or cordycepin nucleotides. Data are from the same experiments as Figs. 1C/1D, and are mean ± SD (n = 3). Asterisks denote statistical significance of differences from incubations without cordycepin (adenine nucleotides) or incubations with 10 µM cordycepin (cordycepin nucleotides).

(D) Amount of cordycepin remaining in the medium of HepG2 cells at various times following its addition to a final concentration of 100 µM. Results, derived from LC:MS analysis, are mean ± SD (n = 3).

Hawley et al Figure S2

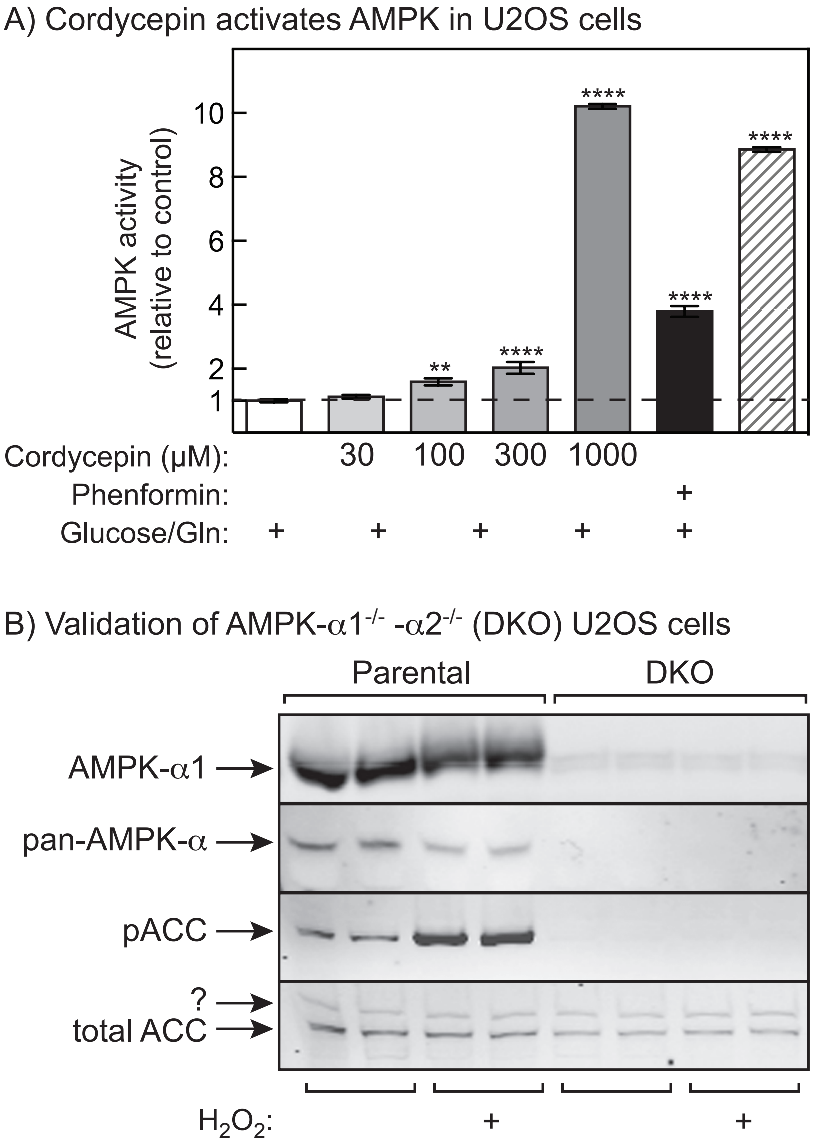

Supplementary Fig. 2 (related to Fig. 2):
(A) Activation of AMPK in U2OS cells by various concentrations of cordycepin, by phenformin (10 mM) or by removal of glucose and glutamine for 1 hr. Results are shown as mean ± SEM (n = 3); asterisks indicate mean values significantly different from control by 1-way ANOVA.
(B) Validation of double AMPK-α knockout in U2OS cells, made using the CRISPR-Cas9 (D10A) system. Pictures show Western blots of duplicate cell samples, some of which had been treated with 1 mM H_2O_2 for 10 min to activate AMPK. Blots were probed using an AMPK-α1-specific antibody, a pan-AMPK-α antibody, a pACC (Ser80) antibody, or streptavidin to detect biotin-containing proteins such as ACC. Attempts to detect AMPK-α2 in these cells by Western blotting were not successful, most likely because of low expression. However, note that the phosphorylation of ACC in response to H_2O_2 was completely eliminated. In addition, we could not detect any wild type AMPK-α1 or -α2 sequences by sequencing of genomic DNA.
